# Congenital peritoneal encapsulation complicated by closed-loop obstruction and bowel ischemia: a case report

**DOI:** 10.1016/j.ijscr.2025.111399

**Published:** 2025-05-04

**Authors:** Majd Oweidat, Samer Abdallah, Haya Taha, Raed Shawawreh

**Affiliations:** a College of Medicine, Hebron University, Hebron, West Bank, Palestine; b Department of Surgery, Princess Alia Hebron Governmental Hospital, Hebron, West Bank, Palestine; c Department of Radiology, Princess Alia Hebron Governmental Hospital, Hebron, West Bank, Palestine

**Keywords:** Congenital peritoneal encapsulation, Small bowel obstruction, Bowel ischemia, Closed-loop obstruction

## Abstract

**Introduction:**

Congenital peritoneal encapsulation (CPE) is a rare developmental anomaly characterized by an accessory peritoneal membrane surrounding the small bowel. Most cases are asymptomatic or present with intermittent abdominal pain. While CPE has been associated with bowel obstruction in rare instances, bowel ischemia requiring resection has not been reported. We present a unique case of CPE complicated by closed-loop obstruction and bowel ischemia.

**Presentation of case:**

A woman in her late 60s with a history of hypertension and type 2 diabetes mellitus presented with a 10-day history of colicky abdominal pain, vomiting, and constipation. She reported recurrent abdominal pain since childhood, worsening over the past decade. Abdominal examination revealed a left lower quadrant mass. Imaging suggested a closed-loop small bowel obstruction. During exploratory laparotomy, a thick peritoneal membrane encapsulating the small bowel was identified. Bowel ischemia was noted, necessitating resection of a 15 cm ileal segment with anastomosis. Histopathology confirmed a stenotic fibrous band with mild serositis. The patient had an uneventful recovery, with complete resolution of symptoms.

**Discussion:**

CPE is often misdiagnosed due to its rarity and non-specific presentation. This case uniquely shows its potential to cause bowel ischemia, an unreported complication.

**Conclusion:**

Surgeons should consider CPE in cases of unexplained bowel obstruction. Early recognition and surgical intervention are crucial in preventing severe complications such as ischemia.

## Highlights

• Congenital peritoneal encapsulation (CPE) is a rare cause of small bowel obstruction.
• This case uniquely presents CPE complicated by closed-loop obstruction and bowel ischemia.
• Surgical exploration remains essential for definitive diagnosis and management.
• Early recognition and intervention can prevent life-threatening complications like bowel ischemia.

## 1 Introduction

The work has been reported in line with the SCARE Guidelines 2023 criteria [1].

Small bowel obstruction (SBO) is one of the most common surgical emergencies, comprising around 20 % of all emergency laparotomies [2]. Most of the cases are caused by adhesions, hernias or neoplasms, where adhesion bands are the most common cause [2].

Congenital peritoneal encapsulation (CPE) is a rare type of developmental anomaly defined by an additional membrane of peritoneum surrounding some or all of the small bowel [3]. There's less than 50 documented cases of CPE in the literature [4]. CPE shows a 5:3 male predominance, with males displaying a bimodal age distribution (20–30 and 60–70 years), whereas females are diagnosed earlier, typically before 30 years [4].

Given its non-specific presentation, CPE often goes misdiagnosed due to its rarity and nonspecific clinical features. While most asymptomatic cases of CPE are discovered incidentally during surgery or autopsy, many patients report intermittent, chronic abdominal pain prior to diagnosis, leading to delays in recognition [4]. According to the literature, CPE has led to acute bowel obstruction in fewer than 14 cases, and it has never resulted in bowel ischemia requiring resection.

Herein, we report a case of CPE that was diagnosed by direct visualization during surgery, manifesting as chronic intermittent bowel obstruction, complicated by bowel ischemia, and managed by surgical resection.

## 2 Presentation of case

A female patient in her late 60s, with a history of primary hypertension and type 2 diabetes mellitus without complications, presented to our emergency department with a complaint of colicky abdominal pain persisting for ten days. The pain was localized to the left lower abdomen, episodic in nature, and was relieved by vomiting. It was not related to food intake and had no radiation. She reported a history of similar episodic attacks dating back to childhood, with a significant increase in severity over the last decade. The pain was accompanied by frequent vomiting—up to ten episodes per day during acute attacks—initially consisting of food particles, progressing to yellowish, then brown, with a foul odor. Three months prior to presentation, she had an episode of hematemesis requiring hospital admission. She also experienced constipation for the last ten days, associated with abdominal distention, though she was still able to pass flatus.

Her medical history was notable for essential hypertension and type 2 diabetes mellitus, managed with *Bisoprolol*, *Losartan Potassium*, *Metformin*, *Gliclazide*, *Aspirin*, and *Famotidine*. A gastroscopy performed a decade earlier had been unremarkable. There was no history of food or drug allergies, previous blood transfusions, or similar conditions among family members. A recent hospitalization occurred a few months prior due to colitis and coffee ground vomiting.

On examination, she appeared alert, conscious, and oriented, with an average build and stable vital signs, except a blood pressure of 160/65 mmHg. There were no signs of jaundice, pallor, or cyanosis. Abdominal examination revealed a soft, lax abdomen with a deep palpable mass in the left lower quadrant. There were no peritoneal signs, but deep palpation revealed tenderness in the left lower quadrant, and percussion showed a dull area over the same region. Bowel sounds were loud, sharp, and frequent, suggestive of obstructive pathology. A digital rectal examination revealed an empty rectum without palpable masses.

Laboratory investigations shown in Table 1 revealed mild hypokalemia (potassium = 3.3 mmol/L) and a slightly elevated alanine aminotransferase (ALT = 51 U/L). Other laboratory parameters were unremarkable.

**Table 1:** Laboratory investigations done for the patient presented in this article.

Test	Result	Reference range
Hemoglobin (HGB)	13.9 g/dL	12–15.5 g/dL
White blood cell count (WBC)	9.4 × 10^3^/mm	5–10 × 10^3^/mm
Platelets	262 × 10^3^/μL	150–400 × 10^3^/μL
Mean corpuscular volume (MCV)	77.3 fL	80–98 fL
Mean corpuscular hemoglobin (MCH)	25.1 pg	28–32 pg
Creatinine	0.7 mg/dL	0.5–1.1 mg/dL
Blood urea nitrogen (BUN)	13 mg/dL	10–20 mg/dL
Sodium (Na)	140 mmol/L	135–147 mmol/L
Potassium (K)	3.3 mEq/L	3.5–5 mEq/L
Chloride (Cl)	107 mmol/L	95–105 mmol/L
Aspartate aminotransferase (AST)	37 U/L	0–35 U/L
Alanine aminotransferase (ALT)	51 U/L	10–40 U/L
Thyroid-stimulating hormone (TSH)	1.3 μU/mL	0.5–4.0 μU/mL
Amylase	47 U/L	25–125 U/L
Urine pH	Acidic	4.5–7.8
Urine protein	Negative	Negative
Urine ketones	+3	Negative
Urine WBCs	4–6 HPF	0–5 HPF
Urine RBCs	3–5 HPF	0–5 HPF

**Fig. 1:**
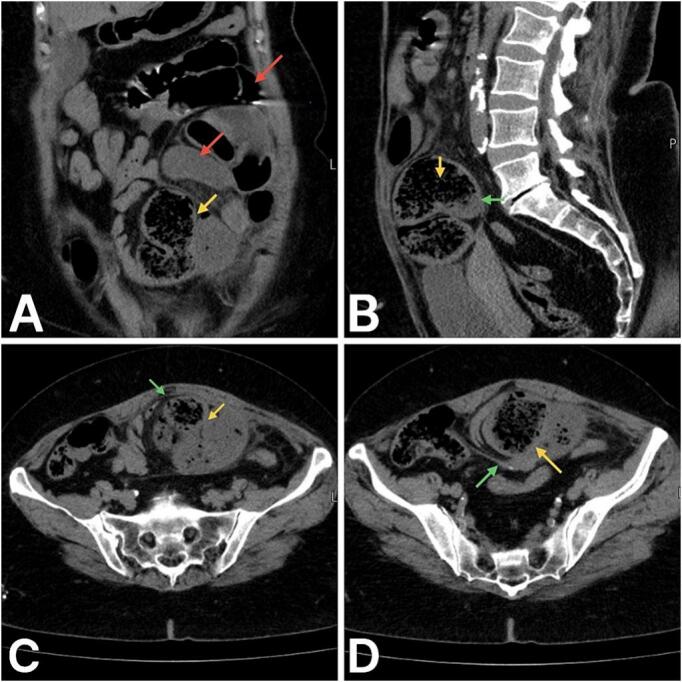
Abdominal CT without IV contrast. A: Coronal image shows proximal dilated small bowel loops (red arrows) and the encapsulated C-shaped configuration of the closed-loop (yellow arrows). B, C, and D are sagittal, sagittal and axial sections, respectively, which show a thin layer of the peritoneum (green arrow) surrounding the closed loop (yellow arrow). (For interpretation of the references to colour in this figure legend, the reader is referred to the web version of this article.)

**Fig. 2:**
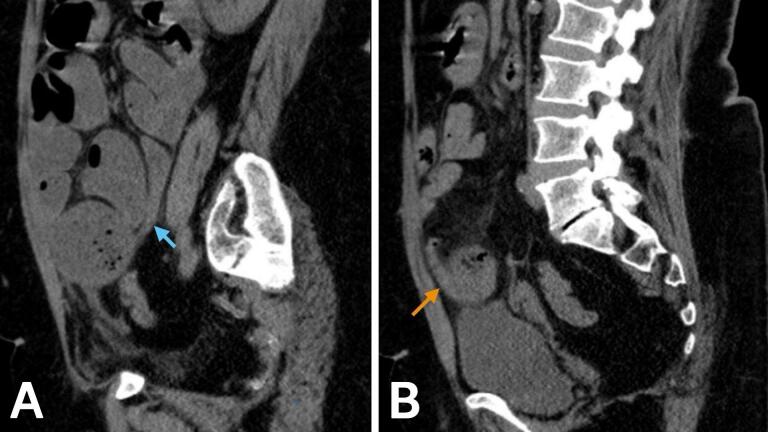
Sagittal abdominal CT without IV contrast. Both images A and B show tapering involving both ends of the closed loop, representing a double beak sign. The proximal and distal limbs are indicated by the blue and orange arrows, respectively. (For interpretation of the references to colour in this figure legend, the reader is referred to the web version of this article.)

Imaging studies including abdominal radiographs and computed tomography (CT), were performed to evaluate the underlying etiology. The abdomen standing X-ray revealed multiple air-fluid levels, and an abdomen CT without contrast was ordered to rule out intestinal obstruction. The CT images shown in Fig. 1 and Fig. 2 showed dilated small bowel loops just proximal to a transition area of encapsulated small bowel loops “closed-loop” at the level of the supravesical fossa. The closed loop was dilated and exhibited a small bowel feces sign. This area was surrounded by fat stranding. No free fluid in the pelvis or abdomen was seen. Therefore, the presumed diagnosis of closed-loop small bowel obstruction due to internal hernia was made.

**Fig. 3:**
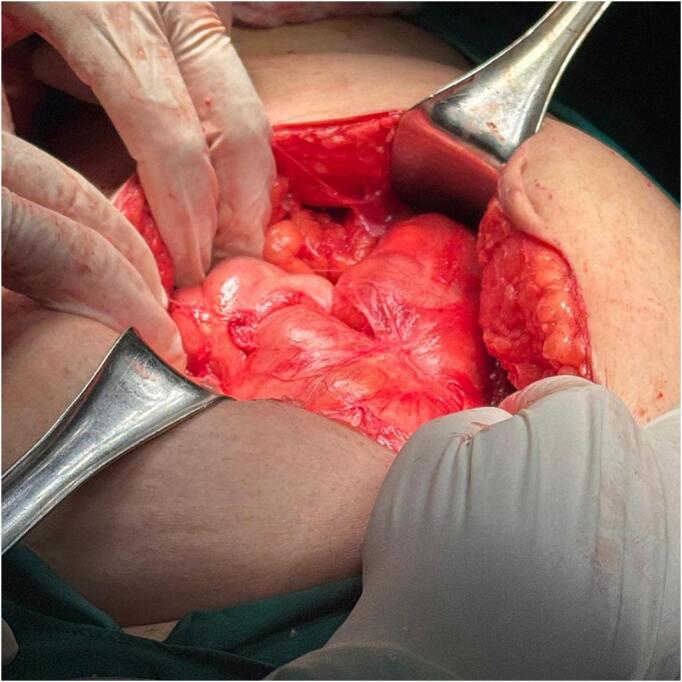
Intraoperative findings showing peritoneal encapsulation of the small bowel with proximal dilation due to closed-loop obstruction.

The patient underwent an exploratory laparotomy, with a midline incision followed by opening layer by layer until the peritoneal cavity was reached. The entire small bowel was encased by thick multiple peritoneal reflections with adhesions between small bowel loops (Fig. 3). Initially, normal anatomy was difficult to identify. Gentle adhesiolysis was performed to separate the adhesions while preserving the integrity of the small bowel wall. Upon opening the peritoneal sac, the small bowel was identified without evidence of a mass. As the anatomy became clearer, the small bowel loops were found to be gangrenous and nonviable, with a transitional zone in the ileum formed by a closed-loop obstruction at two points approximately 5 cm apart. This obstruction led to proximal bowel dilation, which was responsible for the patient's condition. The affected bowel segment displayed a clear demarcation line and remained nonviable despite the application of warm saline. Approximately 15 cm of the ileum, located 100 cm proximal to the ileocecal valve, was resected. The affected ileal loop was transected, transposed, and a side-to-side anastomosis was performed using a linear stapler. A second layer of interrupted absorbable sutures was applied to secure the stapled anastomosis. The mesentery was closed, the abdominal cavity was washed with clear saline, and a pelvic drain was placed. The excised specimen was sent for histopathological examination.

The histopathological analysis confirmed the presence of a stenotic fibrous band with mild serositis in the terminal ileum, without evidence of granulomas, parasites, dysplasia, or malignancy. The post-operative course was uneventful, with no immediate complications. The patient was discharged with a follow-up plan, which showed no abnormal findings. At her follow-up visits, she reported a complete resolution of her chronic symptoms and was able to resume her normal daily activities without recurrence of abdominal pain, vomiting, or constipation. Long-term surveillance was advised due to the rare nature of her condition, and she was instructed to seek immediate medical attention if symptoms recurred.

## 3 Discussion

In this article, we present a case of CPE with a long history of recurrent, episodic abdominal pain and vomiting, who presented with acute-on-chronic intestinal obstruction. Surgical exploration revealed a peritoneal encapsulation of the small bowel with a closed-loop obstruction and ischemic changes, necessitating bowel resection and anastomosis.

The embryological basis of CPE is yet to be made clear, though it has been suggested that unusual adhesion of peritoneal layers during midgut herniation between weeks 8–10 of gestation may lead to the development of an accessory sac around the small bowel [3]. The most accepted theory, proposed by *Papez* in 1932, suggests that aberrant peritoneal adhesion between the umbilical hernia lining and the caudal duodenum results in the formation of an accessory peritoneal sac [5]. Another theory by *Thorlakson* et al. attributes CPE to abnormal midgut reduction, leading to peritoneal encapsulation of the small bowel [6]. The membrane that forms may hinder the movement of the bowel, predisposing the patient to recurrent episodes of subacute obstruction, which may later lead to closed-loop obstruction [4]. Despite these hypotheses, CPE's rarity and lack of associated mesenteric anomalies make its pathogenesis challenging to fully elucidate.

There is no gold-standard test for diagnosing CPE, making preoperative identification challenging. However, literature suggests that a combination of clinical presentation, abdominal X-ray, CT and histopathology can aid in diagnosis [4]. Patients typically present with abdominal pain, nausea, and vomiting, with intermittent chronic symptoms sometimes preceding acute episodes [4]. In this case, the preoperative differential diagnosis included internal supravesical hernia, given its higher incidence and overlapping radiological features with CPE in closed-loop obstruction. Internal hernias, involving viscera protrusion through peritoneal defects, account for up to 5.8 % of small bowel obstructions and show encapsulated and distended bowel loops [7].

Among imaging modalities, CT can provide valuable clues, with findings such as a thin peritoneal membrane encapsulating clustered, dilated small bowel loops in an atypical location for an internal hernia [8]. This feature, if identified, helps differentiate CPE from encapsulating peritoneal sclerosis (abdominal cocoon), which has a thicker fibrocollagenous peritoneal sac, inflammatory changes, and a history of peritoneal dialysis, which were absent in this case [9]. However, in many cases, the peritoneal sac is not clearly visible on imaging, with only 40 % of documented cases reporting radiological evidence of a membranous capsule surrounding the bowel [9]. Hence, CT scanning may not have the resolution to visualize the accessory sac in all cases and should remain only an adjunct to clinical diagnosis. Ultimately, the diagnosis is often confirmed intraoperatively when the accessory membrane is directly observed during laparotomy.

The management of CPE typically involves surgical intervention. It generally includes laparotomy, excision of the peritoneal sac, and careful dissection to avoid damaging the mesenteric vasculature. Prognosis is typically excellent following prompt surgery [4,10].

We highlight by this case that CPE, though rare, can lead to severe complications such as bowel ischemia, a finding not previously documented in the literature. Physicians should consider CPE in the differential diagnosis of unexplained abdominal pain and intermittent bowel obstruction, particularly when imaging suggests encapsulated small bowel loops. As early recognition and surgical intervention can prevent life-threatening outcomes like ischemia.

## 4 Conclusion

This case highlights the importance of considering CPE in patients with bowel obstruction. Unlike previously reported cases, our patient developed bowel ischemia, highlighting a severe complication not previously documented. Physicians should recognize CPE's potential for ischemia and consider timely surgical intervention to prevent life-threatening outcomes.

## Consent

Written informed consent was obtained from the patient for publication and any accompanying images. A copy of the written consent is available for review by the Editor-in-Chief of this journal on request.

## Ethical approval

Local ethical committees don't require ethical approval for reporting individual cases.

## Guarantor

Dr. Raed Shawawreh & Dr. Samer Abdallah.

## Registration of research studies

Not applicable.

## Funding

None.

## Author contribution

*M.O.:* Provided conceptualization, data curation, writing – original draft preparation, writing – review & editing, investigation, visualization, validation, software and resources.

*S.A.:* Provided conceptualization, writing – original draft preparation, investigation, visualization and methodology.

H.T.M.T: Provided conceptualization, writing – original draft preparation, investigation, visualization and methodology.

*R.S:* Provided supervision.

## Declaration of competing interest

None.
